# Co-stimulation of LPAR_1_ and S1PR_1/3_ increases the transplantation efficacy of human mesenchymal stem cells in drug-induced and alcoholic liver diseases

**DOI:** 10.1186/s13287-018-0860-y

**Published:** 2018-06-14

**Authors:** Mianhuan Li, Yi Lv, Feng Chen, Xiaoyan Wang, Jiang Zhu, Hao Li, Jia Xiao

**Affiliations:** 1 Department of Gastroenterology, Clinical Medicine Research Institute, The First Affiliated Hospital of Jinan University, Guangzhou, 510632 People’s Republic of China; 2 State Key Discipline of Infectious Diseases, Department of Infectious Diseases, Shenzhen Third People’s Hospital, Shenzhen, 518112 People’s Republic of China; 3 JM Medical (Shenzhen), LLC, Shenzhen, Shenzhen, 518000 People’s Republic of China; 4 Department of Head and Neck Surgery, State Key Laboratory of Oncology in South China, Sun Yat-sen University Cancer Centre, Guangzhou, 510060 People’s Republic of China; 5 School of Biomedical Sciences, The University of Hong Kong, Hong Kong, Hong Kong, Special Administrative Region of China

**Keywords:** Stem cell therapy, LPA, S1P, Transplantation efficacy

## Abstract

**Background:**

One of the major obstacles facing stem cell therapy is the limited number of functional stem cells available after transplantation due to the harsh microenvironment surrounding the damaged tissue. The aim of this study was to delineate the mechanistic involvement of lysophosphatidic acid receptors (LPARs) and sphingosine-1-phosphate receptors (S1PRs) in the regulation of anti-stress and transplantation efficacy of stem cells.

**Methods:**

Human adipose-derived mesenchymal stem cells (hADMSCs) were treated with chemical toxin or ethanol to induce cell stress. Lysophosphatidic acid (LPA) and/or sphingosine-1-phosphate (S1P) were co-treated to examine their protective effects and mechanisms on stem cell damage. Acute liver failure and alcoholic liver disease murine models were also established to test the transplantation efficacy of hADMSCs with or without LPA/S1P pre-incubation.

**Results:**

Co-stimulation of LPAR_1_ by LPA and S1PR_1/3_ by S1P synergistically enhanced the anti-stress ability of hADMSCs induced by chemical or ethanol incubation in vitro. Downstream pathways involved in this process included the Gi protein (but not the G_12/13_ proteins), the RAS/ERK pathway, and the PI3K/Akt pathway. Upon cell injury, the nuclear translocation of nuclear factor-kappa B (NF-κB) was promoted to facilitate the activation of downstream pro-inflammatory gene transcription, which was ameliorated by co-treatment with LPA and/or S1P. Increased secretion of interleukin (IL)-10 from stem cells by LPA and/or S1P seemed to be one of the major protective mechanisms since blocking IL-10 expression significantly aggravated stress-induced cell damage. In a drug-induced acute liver failure model and a chronic alcoholic liver disease model, pre-conditioning with LPA and/or S1P significantly enhanced the survival ratio and the therapeutic efficacy of hADMSCs in mice, including ameliorating histological damage, oxidative stress, inflammation, fibrosis, lipid metabolism dysfunction, and enhancing alcohol metabolizing enzyme activity. Importantly, supplementing LPA and/or S1P did not alter the basic characteristics of the hADMSCs nor induce tumour formation after cell transplantation.

**Conclusions:**

Co-use of LPA and S1P represents a novel and safe strategy to enhance stem cell transplantation efficacy for future drug- and alcoholic-related liver disease therapies.

**Electronic supplementary material:**

The online version of this article (10.1186/s13287-018-0860-y) contains supplementary material, which is available to authorized users.

## Background

Drug-induced and alcoholic liver diseases are common but severe clinical problems worldwide. For example, drug-induced liver injury (DILI) occurs between 10 and 15 per 10,000 to 100,000 persons exposed to prescription medications annually and accounts for approximately 10% of all cases of acute hepatitis [1, 2]. In the US, 15.1 million adults are reported to have an alcohol use disorder, including 9.8 million men and 5.3 million women. An estimated 88,000 people die from alcohol-caused disease annually [3]. When excessive drugs/alcohol are consumed, the hepatic metabolizing system fails to detoxify them, and subsequent inflammation and oxidative stress may induce liver failure which warrants timely liver transplantation. Due to the rapid progress of regenerative medicine, stem cell-based transplantation has become a promising strategy to cover shortages in liver transplantation availability due to insufficient donor organs, rejection, and infection [4, 5].

The high death rate of stem cells post-transplantation is one of the major problems in clinical therapy. This phenomenon is primarily due to the harsh inflammatory and oxidative stress environment at the site of the injury [6]. It has been demonstrated that pre-conditioning of antioxidants in the culture medium of stem cells could significantly enhance the cell resistance to oxidative stress/inflammation and the transplantation efficacy in several disease models, including edaravone in an acute liver failure model [7] and N-acetylcysteine in a myocardial infarction model [8]. However, the exact mechanisms of antioxidant-mediated cell protection, particularly the direct interacting receptors of an agent on the cell membrane, remain largely unknown.

Lysophosphatidic acid (LPA) is a well-characterized lipid-derived ligand that regulates cell growth, differentiation, and motility in various cell types. Stimulation of LPA receptors (LPARs) by LPA is reported to elicit hepatocyte proliferation and to protect the liver from acute injury [9]. Sphingosine-1-phosphate (S1P) is also a potent bioactive molecule for basic cell function regulations through a family of five G protein-coupled receptors referred to as S1P receptor (S1PR) types 1–5 [10]. A recent study found that activation of the S1P/S1PR pathway was able to promote liver fibrosis-associated angiogenesis [11]. Moreover, although several reports demonstrated the protective effects of LPA and S1P on stress-induced stem cell injury [12–14], whether and how the modulation of the LPA/S1P pathways influence the anti-stress phenotypes and transplantation efficacy in the injured liver of stem cells are poorly understood. In the current study, we demonstrated that the concurrent stimulation of LPAR_1_ and S1PR_1/3_ synergistically enhanced the anti-stress ability of human adipose-derived mesenchymal stem cells (hADMSCs) in vitro and enhanced the transplantation therapeutic efficacy in a drug-induced acute liver failure and a chronic alcoholic liver disease (ALD) model in vivo. The RAS/ERK, PI3K/Akt, and nuclear factor-kappa B (NF-κB)-interleukin (IL)-10) axis were involved in this protection.

## Methods

### Cells, chemicals, and reagents

All cell culture reagents and consumables were obtained from Gibco (Carlsbad, CA, USA) or Corning Incorporated (Corning, NY, USA). hADMSCs (from a single donor) were purchased from Cyagen Biosciences (Guangzhou, China). All immunophenotypes of the hADMSCs were validated by the manufacturer. d-galactosamine (Gal), lipopolysaccharide (LPS), methylthiazolyldiphenyl tetrazolium bromide (MTT), pertussis toxin (PTX), W146, JTE013, salirasib, and MK-2206 were purchased from Sigma-Aldrich (St. Louis, MO, USA). AM966 and CAY10444 were obtained from APExBIO (Houston, TX, USA) and Cayman Chemical (Ann Arbor, MI, USA), respectively. The NF-κB p65 acetylation inhibitor anacardic acid (AnaAcid) was a product of Abcam (Cambridge, UK). All antibodies and UO126/wortmannin were purchased from Cell Signalling (Danvers, MA, USA).

### Stem cell treatments

hADMSCs were cultured in Dulbecco’s modified Eagle’s medium (DMEM)/F12 medium with 10% (v/v) foetal bovine serum (FBS) at 37 °C with 5% CO_2_ using a cell incubator (ThermoFisher Scientific, Waltham, MA, USA). To induce severe cellular oxidative stress and inflammatory responses, stem cells were treated with 0.1 μg/ml LPS and 200 μM H_2_O_2_ simultaneously for 24 h [7] or 400 mM ethanol for 24 h [15]. For the knockdown of stem cell endogenous IL-10/G_12/13_ expression, corresponding MISSION shRNAs (Sigma-Aldrich) were transfected into hADMSCs using the Lipofectamine 3000 reagent system (Invitrogen, Carlsbad, CA, USA). Knockdown efficiency (after 48 h) was verified according to the instructions from the manufacturer.

### Cell viability test

Stem cell viability changes were measured with the MTT assay. After treatments, cells were washed three times in sterile phosphate-buffered saline (PBS) and later incubated with 5 mg/ml MTT for 4 h. The cells were subsequently dissolved in dimethyl sulphoxide (DMSO; Sigma-Aldrich). The absorbance of MTT was measured at 570 nm, and pure DMSO was used as the blank.

### Flow cytometry

Stem cell apoptosis was quantified using an Annexin V/FITC kit from Beyotime Biotechnology according to the manufacturer’s instructions (Jiangsu, China) in a BD FACScalibur machine (Beckman Coulter, Brea, CA, USA). This assay discriminates between intact (Annexin V^−^/PI^−^), early apoptotic (Annexin V^+^/PI^−^), late apoptotic (Annexin V^+^/PI^+^), and necrotic (Annexin V^−^/PI^+^) cells as analysed by the FlowJo flow cytometry software. Approximately 2 × 10^4^ cells were analysed in each sample.

### Caspase-3/7 activity assay

The measurement of caspase-3/7 activity changes in cell lysates or tissue-extracted proteins was performed with a Cell Meter Caspase 3/7 Activity Apoptosis Assay Kit (AAT Bio., Sunnyvale, CA, USA) according to the user manual. The results were read at 520 nm in a micro-plate reader (Bio-Rad, Hercules, CA, USA) and expressed as fold changes in caspase-3/7 activity relative to the control.

### Immunofluorescence assay

To measure the production of cellular free radicals, stem cells cultured on 12-mm round glass cover slips were fixed with 4% formaldehyde (v/v) at room temperature for 15 min and then permeabilized with 1% Triton X-100 in Tris buffer (Gibco) for another 15 min. To block non-specific staining, the cells were treated with PBS buffer containing 5% bovine serum albumin (BSA) for 1 h at 37 °C. Subsequently, the cells were incubated in the same solution for 2 h at room temperature with primary antibodies against 5,5-dimethyl-1-pyrroline N-oxide (DMPO; 1:100, Rockland, Limerick, PA, USA). After three washes with PBS buffer, the cells were incubated for 1 h with goat antibody against mouse IgG conjugated with FITC (1:1000, Abcam) at room temperature. To show the nuclei, the cells were counter-stained with Hoechst 33,342 (Beyotime) for 15 min at room temperature. The slides were mounted with a fluorescent mounting medium (KPL, Gaithersburg, MD, USA) before examination under an inverted fluorescence microscope (IX71; Olympus microscope, Tokyo, Japan).

### GSH/GSSG ratio measurements

The ratio between the reduced glutathione (GSH) and the oxidized glutathione (GSSG) of stem cell lysates was measured to determine the change in endogenous antioxidant level using a GSH/GSSG detection assay kit (Abcam).

### Semi-quantitative and quantitative real-time polymerase chain reaction

Total RNA was extracted from the stem cells using the illustra™ RNAspin mini kit (GE healthcare, Amersham, UK) and the cDNA was generated from 2 μg total RNA using the SuperScript™ First-Strand Synthesis System (Invitrogen). To analyse the LPAR and S1PR subtype expressions in stem cells, the following gene-specific primers were used: LPAR_1_: 5’-TCTTCTGGGCCATTTTCAA-3′ and 5’-GCCGTTGGGGTTCTCGTT-3′; LPAR_2_: 5’-CCTACCTCTTCCTCATGTTC-3′ and 5’-AATGATGACAACCGTCTTGACTA-3′; LPAR_3_: 5’-TGTCAACCGCTGGCTTCT-3′ and 5’-CAGTCATCACCGTCTCATTAG-3′; S1PR_1_: 5′- TCTGCTGGCAAATTCAAGCGA-3′ and 5’-GTTGTCCCCTTCGTCTTTCTG-3′; S1PR_2_: 5’-CATCGTCATCCTCTGTTGCG-3′ and 5’-GCCTGCCAGTAGATCGGAG-3′; S1PR_3_: 5’-CGGCATCGCTTACAAGGTCAA-3′ and 5’-GCCACGAACATACTGCCCT-3′. Parallel amplification of glyceraldehyde-3-phosphate dehydrogenase (GAPDH) with 5’-CTGGGCTACACTGAGCACC-3′ and 5’-AAGTGGTCGTTGAGGGCAATG-3′ was used as the internal control. Polymerase chain reaction (PCR) products were run and imaged on 1.2% agarose gels stained with ethidium bromide (Sigma-Aldrich). cDNA templates reverse transcribed from neonatal rat cardiac myocytes were used as positive controls for each set of primers [16].

To analyse the expressional changes of key genes during stem cell differentiation, synthesized cDNAs from non-induced or differentiated stem cell lysates were subjected to quantitative real-time PCR using the Takara SYBR premix Taq quantitative PCR system (Takara Bio Inc., Shiga, Japan) and a MyiQ2 real-time PCR machine (Bio-Rad) as described previously [17]. All real-time PCR procedures, including the design of primers, validation of PCR environment, and quantification methods, were performed according the MIQE guidelines [18].

### NF-κB p65 nuclear translocation and activity assays

Changes in the NF-κB p65 nuclear translocation status after stress induction and/or chemical protection were evaluated by Western blot using specific p65 antibodies. Quantification of nuclear NF-κB p65 activity was tested using an NF-kB (p65) Transcription Factor Assay Kit (Abcam).

### Murine acute liver failure and chronic alcoholic liver injury models

All animal experiments, including procedures, sampling, and animal care, in the current study were approved by and completed in accordance with the guidelines and regulations of the ethical committee of Shenzhen Third People’s Hospital. Male 6-week-old (~ 20 g) non-obese diabetic severe combined immune-deficient (NOD/SCID) mice were purchased from the Guangdong Experimental Animal Centre (Guangzhou, China). To induce acute liver failure (ALF), mice were intraperitoneally injected with 600 mg/kg Gal and 8 μg/kg LPS dissolved in PBS simultaneously, with or without tail-vein injections with 2 × 10^6^ hADMSCs (passage 2; untreated, 5 μM LPA-pre-treated and/or 0.25 μM S1P-pretreated for 2 h) 6 h later. The dosage combination of Gal and LPS, as well as the delivery route/dose selection of stem cells, were selected based on our previous studies [7, 19]. Mice serum and liver tissues were collected on day 3 after stem cell transplantation since previous studies showed that on day 3 (not too early or too late) samplings could indeed exhibit therapeutic effects from drugs or stem cells [7, 19].

For the induction of chronic alcoholic liver injury, the National Institute on Alcohol Abuse and Alcoholism (NIAAA) model was established as previously described with minor modifications. This model has been shown to produce evident steatosis and mild inflammation/fibrosis during a relatively short period [20, 21]. In brief, NOD/SCID mice were orally fed with the Lieber-DeCarli ethanol liquid diet (5%) ad libitum for 10 days and were then allowed to binge drink 5 g/kg ethanol. Tail vein injections with 2 × 10^6^ hADMSCs (passage 2; untreated, 5 μM LPA-pre-treated and/or 0.25 μM S1P-pretreated for 2 h) were performed at day 3 and day 9 after the onset of ethanol consumption. We tested three doses of stem cell injections (5 × 10^6^, 1 × 10^6^, and 2 × 10^6^) in the pilot study and observed that a 2 × 10^6^ single injection generated the best outcome. Moreover, the therapeutic stem cell injection times were optimized by our pilot studies (data not shown). Mice serum and liver tissue were collected 9 h after the binge drinking (which was the endpoint of the entire experiment).

### Serum and liver tissue analysis

Mice serum alanine aminotransferase (ALT) and aspartate aminotransferase (AST) levels were measured using ALT (SGPT) and AST (SGOT) reagent sets (Teco Diagnostics, Anaheim, CA, USA) according to the manufacturer’s instructions. Liver tissue samples were fixed in 10% phosphate-buffered formalin, processed for histology, and embedded in paraffin blocks. Five-micrometre tissue sections were cut and stained with haematoxylin/eosin (H&E) or Sirius Red. The NAFLD activity score (NAS) system was applied to evaluate the alcoholic liver injury in the NIAAA model [22].

### Stem cell transplantation efficacy test and albumin immunohistochemistry

To quantify the transplanted hADMSCs that homed to the mice livers, a human Down syndrome sequence-based real-time PCR quantification system was used in the current study [7]. In brief, hepatic genomic DNA samples were extracted using a QIAamp genomic DNA extraction kit (Qiagen, Hilden, Germany). A pair of primers (5′- ATGCTGATGTCTGGGTAGGGTG-3′ and 5’-TGAGTCAGGAGCCAGCGTATG-3′) that generate a 141-bp fragment of human Down syndrome region at chromosome 21 was used to quantify the human-derived cells. To verify the quantitative PCR results, we performed an immunohistochemistry (IHC) assay using human albumin antibody in sectioned mice liver samples.

### Western blotting, ELISA, and hepatic key gene PCR assays

Protein extraction/quantification from cell or liver tissue, as well as the Western blotting assay, were conducted as previously described [7]. Parallel blotting of GAPDH was used as the internal control. Culture medium tumour necrosis factor (TNF)-α/IL-6/IL-10 levels and mice serum TNF-α levels were measured using corresponding enzyme-linked immunosorbent assay (ELISA) kits from PeproTech (Rocky Hill, NJ, USA) according to the manufacturer’s instructions. Hepatic expressional changes of oncostatin M (OSM), sterol regulatory element-binding protein (SREBP)-1c, and transforming growth factor (TGF)-β were measured using the EIA kits as previously described [7, 23].

### Aldehyde dehydrogenase (ALDH)2 and malondialdehyde (MDA) assays

Hepatic ALDH2 activity changes after alcoholic liver injury and/or stem cell transplantation were measured using an ALDH2 activity assay kit (Abcam). Levels of the end-product of lipid peroxidation (MDA) in the liver tissue samples were determined using a Bioxytech LPO-586™ kit (Oxis Research, Portland, OR, USA). The reaction products were measured spectrophotometrically at 586 nm.

### In vitro differentiation and in vivo transplantation safety assays

The effects of LPA and/or S1P (constantly dissolved in the culture medium throughout the induction period) on stem cell adipogenic or osteogenic differentiation abilities were studied as described previously using the corresponding kits from Saliai Biotechnology (Guangzhou, China) [19]. Briefly, passage 3 hADMSCs were seeded at a density of 1.5 × 10^4^ cells/cm^2^ on 6- or 24-well plates with expansion medium supplemented with 1 mM DEX, 10 mg/l insulin, and 0.5 mM IBMX (all from Sigma) for 4 weeks to induce adipogenic phenotypes. Culture medium containing 5 μM LPA and/or 0.25 μM S1P was refreshed every 3 days. To induce the osteogenic phenotypes, passage 3 hADMSCs were seeded at a density of 3 × 10^4^ cells/cm^2^ on 6- or 24-well plates with expansion medium supplemented with 0.1 mM dexamethasone, 10 mM β-glycerophosphate, and 50 mg/ml vitamin C (all from Sigma) for 4 weeks. Culture medium containing 5 μM LPA and/or 0.25 μM S1P was refreshed every 3 days. Adipogenic and osteogenic differentiations were characterized by Oil Red O staining and Alizarin Red visualizations, respectively.

To ensure the transplantation safety of naive and LPA/S1P-treated MSCs in healthy, ALF, and ALD NOD/SCID mice, we performed a 24-week tumourigenicity study as previously described [24]. Briefly, healthy 6-week-old NOD/SCID male mice (or ALF mice with 600 mg/kg Gal and 8 μg/kg LPS, or ALD mice with the induction using the NIAAA model) received 1 × 10^7^ MRC-5 (negative control), 1 × 10^7^ ES-D3 (positive control), or 1 × 10^7^ MSCs (MSC group, with or without LPA/S1P co-treatment) (12 mice per group for healthy or ALD groups, 18 mice for ALF groups). After 24 weeks or when the mice exhibited severe symptoms of dyspnoea and minimal activity, mice were sacrificed to observe the tumour formation.

### Statistical analysis

Data from each group are expressed as the means ± SEM. Statistical comparisons between groups were performed using the Kruskal–Wallis test followed by Dunn’s post-hoc test to detect differences in all groups. A value of *p* < 0.05 was considered to be statistically significant (Prism 5.0, Graphpad software, Inc., San Diego, CA, USA).

## Results

### Addition of LPA/S1P to the culture medium rescued hADMSCs from cell death

**Fig. 1:**
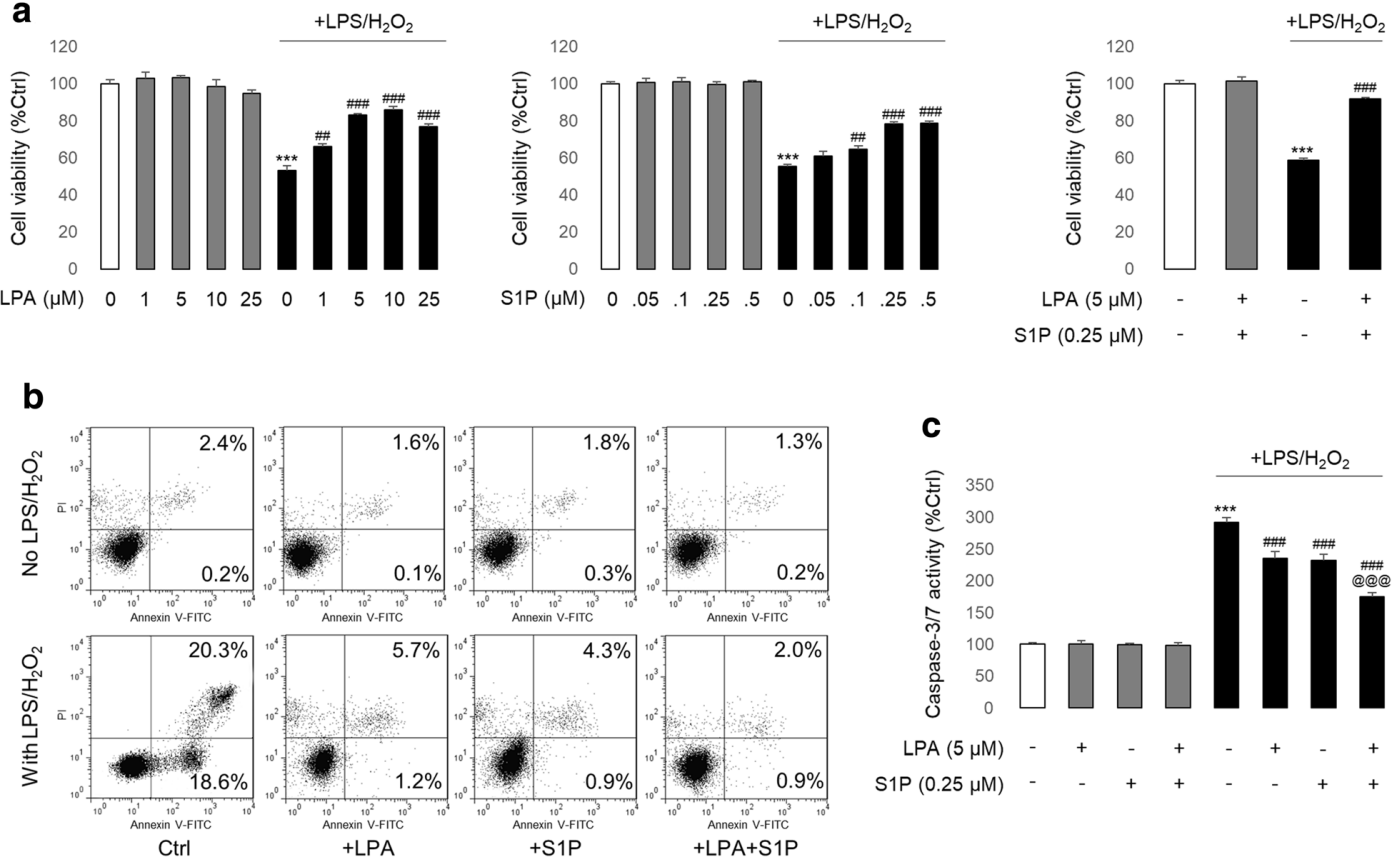
LPA and/or S1P attenuates LPS/H_2_O_2_-induced stem cell death. **a** Changes in hADMSC viability after LPS/H_2_O_2_ intoxification in the presence or absence of LPA/S1P co-treatments (*n* = 4). **b** Distribution of hADMSC apoptotic fractions measured by flow cytometry. Upper right: late phase apoptotic ratio; Lower right: early phase apoptotic ratio. **c** Changes in hADMSC caspase-3/7 activity after LPS/H_2_O_2_ intoxification in the presence or absence of LPA/S1P co-treatments (*n* = 4). Data from each group are expressed as means ± SEM. Statistical comparison between groups was performed with the Kruskal–Wallis test followed by Dunn’s post-hoc test to detect differences in all groups. ***p < 0.001 versus control group; ^##^*p* < 0.01 versus LPS/H_2_O_2_ group; ^###^*p* < 0.001 versus LPS/H_2_O_2_ group; ^@@@^*p* < 0.001 versus LPS/H_2_O_2_ + LPA or S1P group. Ctrl, control; LPA, lysophosphatidic acid; LPS, lipopolysaccharide; S1P, sphingosine-1-phosphate

Incubation of hADMSCs with LPS/H_2_O_2_ for 24 h caused a significant reduction in the cell viability (from 100% to approximately 55%) [7]. The addition of LPA (1, 5, 10, and 25 μM) to the stem cell culture medium 2 h before LPS/H_2_O_2_ exposure recovered cell viability, for which 5 and 10 μM concentrations showed the best recovery effects (from ~ 55% to ~ 85%) (Fig. 1a). Similarly, S1P exhibited evident alleviative effects on damaged stem cells, where 0.25 μM was the optimal treating dosage (from ~ 55% to ~ 80%) (Fig. 1a). Interestingly, the optimal concurrent dose selection experiments for LPA and S1P found that 5 μM LPA and 0.25 μM S1P showed a synergistic protective effect on cell viability relative to LPA or S1P treatment alone (from ~ 80% or ~ 85% versus ~ 94%) (Fig. 1a and Additional file 1: Figure S1). Vehicle-LPA and/or S1P treatments had no obvious adverse impact on stem cell viability (Fig. 1a). In keeping with the cell viability changes, LPA or S1P co-treatment with LPS/H_2_O_2_ significantly ameliorated stem cell apoptosis, which was further demonstrated by the activity changes in cellular caspase-3/7 (Fig. 1b, c). In an ethanol-induced stem cell injury model, the reduction of cell viability and activation of cell apoptosis by ethanol (400 mM) were also ameliorated by co-treatment with LPA or S1P, while concurrent treatment with LPA and S1P showed the best rescuing effects (Fig. 1; Additional file 1: Figures S1 and S2). Thus, we predicted that the addition of LPA and S1P to the culture medium could rescue hADMSCs from drug/ethanol-induced cell death in vitro.

### LPA and S1P protected hADMSCs from toxin/ethanol-induced oxidative stress and inflammatory response

**Fig. 2:**
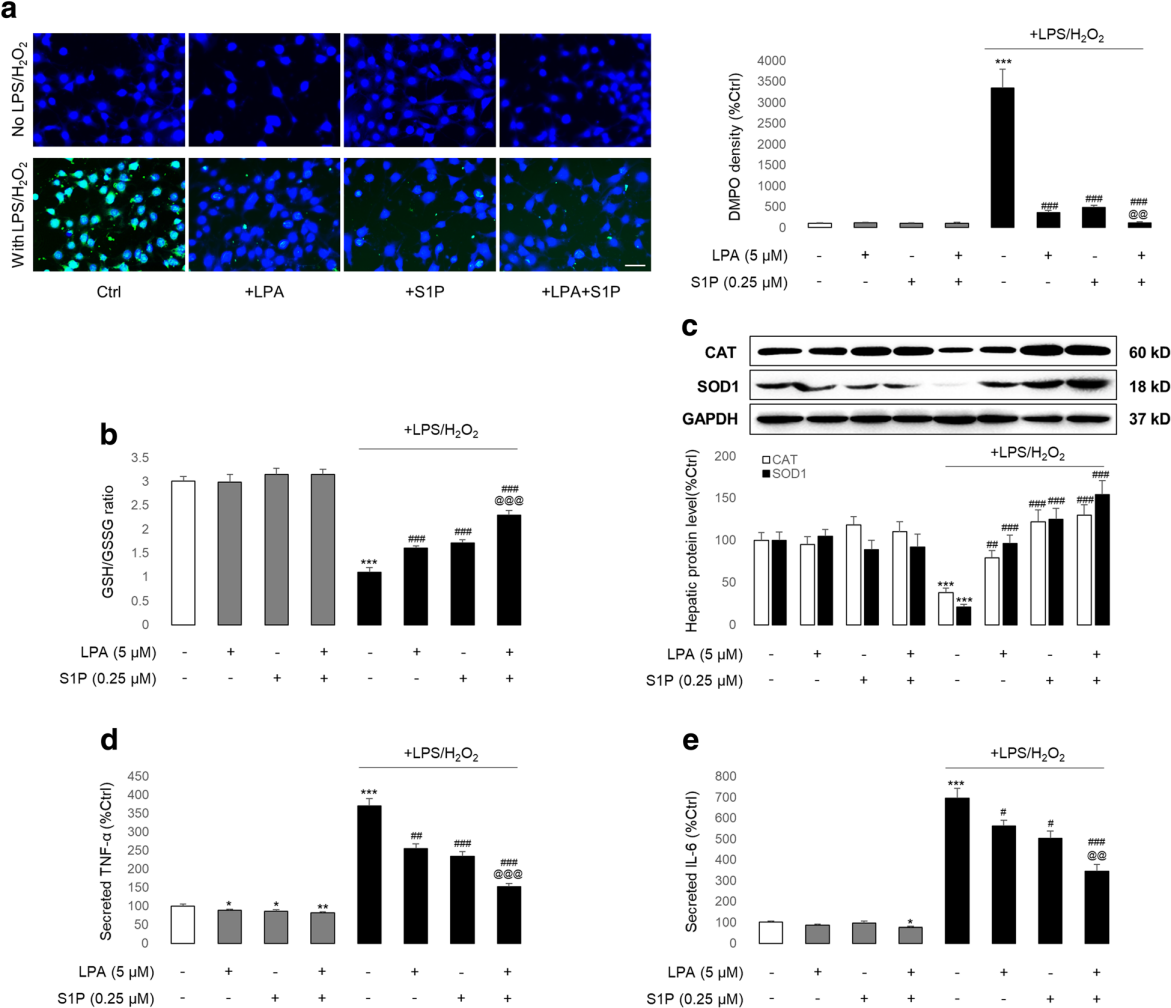
LPA and/or S1P attenuates LPS/H2O2-induced stem cell oxidative stress and inflammation. **a** Representative immunofluorescent image and corresponding quantified data (*n* = 4) of hADMSCs after LPS/H_2_O_2_ intoxification in the presence or absence of LPA/S1P co-treatments (green: DMPO-stained signal; blue: Hoechst 33,342 counter-stained signal). Scale bar = 50 μm. **b** Cellular GSH/GSSG ratio (*n* = 4), **c** cell endogenous antioxidant enzymes (CAT and SOD1) protein level with quantified data (*n* = 3), **d** cell-secreted TNF-α protein level (*n* = 4), and **e** cell-secreted IL-6 protein level changes in hADMSCs after LPS/H_2_O_2_ intoxification in the presence or absence of LPA/S1P co-treatments (*n* = 4). Data from each group are expressed as means ± SEM. Statistical comparison between groups was performed with the Kruskal–Wallis test followed by Dunn’s post-hoc test to detect differences in all groups. ***p < 0.001 versus control group; ^#^*p* < 0.05 versus LPS/H_2_O_2_ group; ^##^*p* < 0.01 versus LPS/H_2_O_2_ group; ^###^*p* < 0.001 versus LPS/H_2_O_2_ group; ^@@^*p* < 0.01 versus LPS/H_2_O_2_ + LPA or S1P group; ^@@@^*p* < 0.001 versus LPS/H_2_O_2_ + LPA or S1P group. CAT, catalase; Ctrl, control; DMPO, 5,5-dimethyl-1-pyrroline-*N*-oxide; GSH, reduced glutathione; GSSG, oxidized glutathione; IL, interleukin; LPA, lysophosphatidic acid; LPS, lipopolysaccharide; S1P, sphingosine-1-phosphate; SOD1, superoxide dismutase 1; TNF, tumour necrosis factor

Since a harsh hepatic environment during drug/ethanol-induced hepatitis progression kills transplanted stem cells because of excessive oxidative stress and inflammatory responses, we used a DMPO antibody to trap biomolecule-centred radicals by forming a new covalent bond with the biomolecule [25]. LPS/H_2_O_2_ or ethanol significantly increased the accumulation of free radicals in hADMSCs, and this was significantly attenuated by co-treatment with LPA and/or S1P. In line with this result, when the ratio of GSH/GSSG was reduced by LPS/H_2_O_2_ or ethanol incubation, LPA and/or S1P addition effectively restored its balance, indicating that the evoked oxidative stress was partially scavenged. Moreover, the restoration of antioxidant enzymes (CAT and SOD1) further demonstrated the antioxidant properties of LPA/S1P treatment (Fig. 2a–c and Additional file 1: Figure S3a, b). Alterations of secreted TNF-α and IL-6 from stem cells after LPS/H_2_O_2_ or ethanol incubation, with or without LPA and/or S1P co-treatment, suggested that cell inflammatory responses could also be attenuated by LPA and S1P supplements (Fig. 2d, e and Additional file 1: Figure S3c, d). It should also be noted that vehicle LPA and/or S1P treatment did not induce oxidative stress or the inflammation of stem cells (Fig. 2 and Additional file 1: Figure S3).

### LPA protects stem cells through an LPAR_1_-Gi pathway while S1P primarily acts through an S1PR_1/3_-Gi pathway

**Fig. 3:**
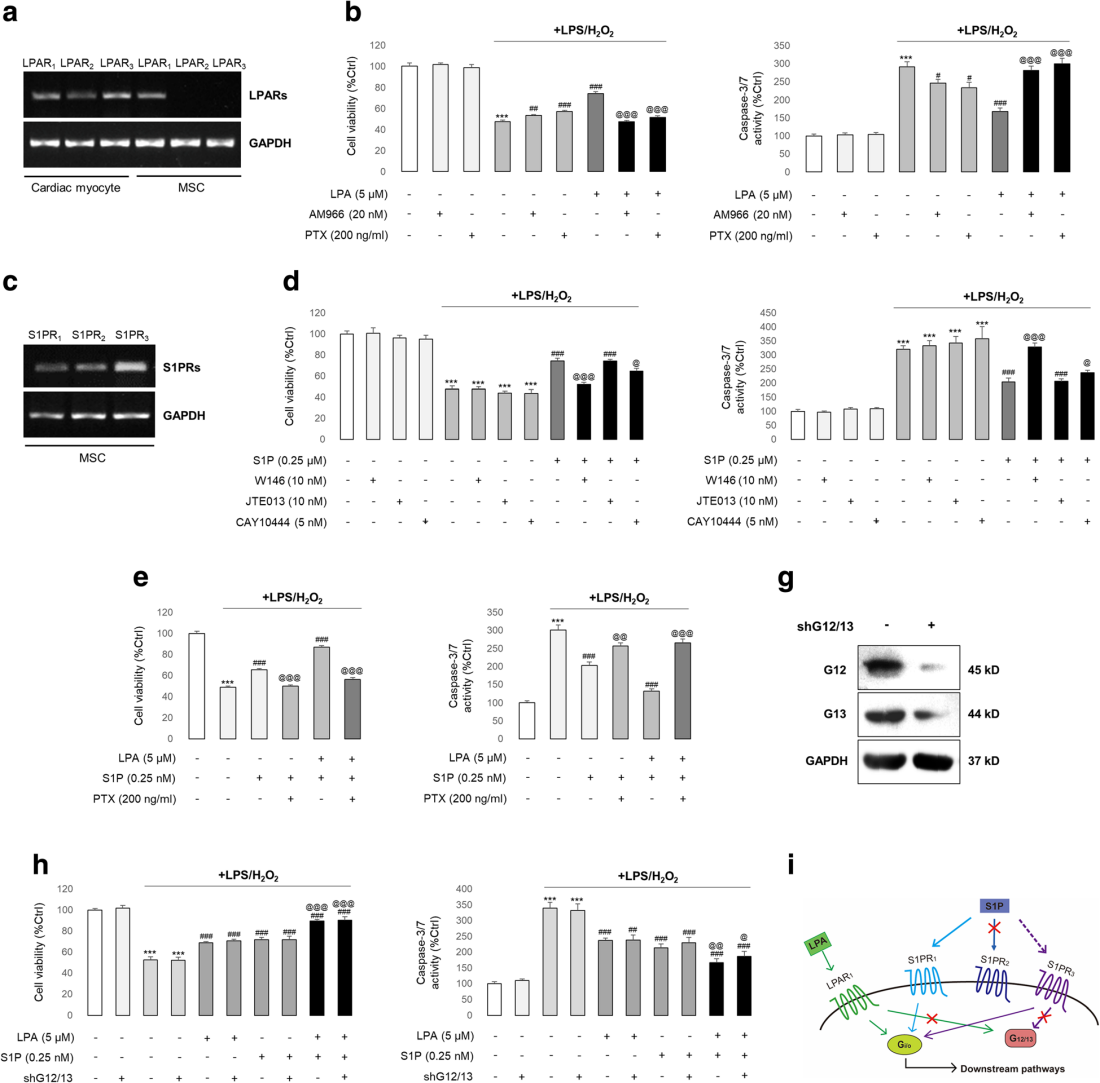
Stimulation of LPAR_1_/Gi by LPA and S1PR_1/3_/Gi by S1P are critical for stem cell protection. **a** The expression profiles of LPAR subtypes were determined in cDNAs from hADMSCs (labelled as MSC) or neonatal rat cardiac myocytes for each set of LPAR primers. **b** Changes in cell viability and caspase-3/7 activity of hADMSCs after LPS/H_2_O_2_/LPA treatments, with or without the AM966 (LPAR_1_ inhibitor), or PTX (Gi inhibitor) co-treatment (*n* = 4). **c** The expression profiles of S1PR subtypes were determined in cDNAs from hADMSCs (labelled as MSC) for each set of S1PR primers (*n* = 4). **d** Changes in cell viability and caspase-3/7 activity of hADMSCs after LPS/H_2_O_2_/S1P treatments, with or without the W146 (S1PR_1_ inhibitor), JTE013 (S1PR_2_ inhibitor), or CAY10444 (S1PR_3_ inhibitor) co-treatment (*n* = 4). **e** Changes in cell viability and caspase-3/7 activity of hADMSCs after LPS/H_2_O_2_/LPA/S1P treatments, with or without the PTX (Gi inhibitor) co-treatment (*n* = 4). **f** Knockdown efficiency verification of G_12/13_ shRNA transfection using G_12_- and G_13_-specific antibodies. **g** Changes in cell viability and caspase-3/7 activity of hADMSCs after LPS/H_2_O_2_/LPA/S1P treatments, with or without the G_12/13_ shRNA co-treatment (*n* = 4). **h** The signalling pathway concluded from this figure. Data from each group are expressed as means ± SEM. Statistical comparison between groups was performed with the Kruskal–Wallis test followed by Dunn’s post-hoc test to detect differences in all groups. ***p < 0.001 versus control group; ^#^*p* < 0.05 versus LPS/H_2_O_2_ group; ^##^*p* < 0.01 versus LPS/H_2_O_2_ group; ^###^*p* < 0.001 versus LPS/H_2_O_2_ group; ^@^*p* < 0.05 versus LPS/H_2_O_2_ + LPA or S1P group; ^@@@^*p* < 0.001 versus LPS/H_2_O_2_ + LPA or S1P group. Ctrl, control; LPA(R), lysophosphatidic acid (receptor); LPS, lipopolysaccharide; S1P(R), sphingosine-1-phosphate (receptor)

To delineate the receptors receiving protective signals from LPA and/or S1P, we first performed RT-PCR analyses of LPARs and S1PRs in hADMSCs. As shown in Fig. 3, LPAR_1–3_ could be detected in the total RNA isolated from cardiac myocytes, which were used as positive controls. In contrast, only LPAR_1_ was detected in the total RNA isolated from the hADMSCs (Fig. 3a). When the LPAR_1_ inhibitor AM966 or the Gi protein inhibitor PTX was added prior to LPS/H_2_O_2_ and LPA incubations, the LPA-mediated cytoprotective effects were reversed. Interestingly, when it was added in the absence of LPA, both AM966 and PTX could partially alleviate LPS/H_2_O_2_-induced stem cell damage (Fig. 3b). This finding was in line with recent reports that antagonism of LPAR_1_ or Gi could protect cells from injuries induced by diabetes [26], hyperoxia [27], and ageing [28]. For S1P, all three subtypes of its receptor were detectable from the stem cell-isolated total RNA (Fig. 3c). Thus, we tested the effects of the supplement of W146 (S1PR_1_ inhibitor), JTE013 (S1PR_2_ inhibitor), or CAY10444 (S1PR_3_ inhibitor) prior to LPS/H_2_O_2_ intoxification, in the absence or presence of S1P co-treatment. It was found that the cytoprotective functions of S1P were abolished by W146, partially impaired by CAY10444, and not influenced by JTE013, indicating that S1PR_1_ and S1PR_3_ were the major receptor subtypes for the signal transduction from S1P treatment (Fig. 3d). Unlike LPA, vehicle S1PR inhibitor treatment did not ameliorate LPS/H_2_O_2_-induced cell damage (Fig. 3d). In addition, S1P- or LPA + S1P-mediated cell protection could also be blocked by PTX, suggesting that the Gi protein was the mutual downstream signal transducer for their protection (Fig. 3e). Since the G_12/13_ protein is also a direct downstream target of both LPAR_1_ and S1PR_3_ [29], we subsequently knocked down its endogenous expression using G_12/13_ shRNA (Fig. 3f) to see whether it was indispensable for LPA/S1P-mediated stem cell protection. We found that the inhibition of G_12/13_ neither influenced the basal cell condition nor interfered with the damage induced by LPS/H_2_O_2_ or protection from LPA/S1P (Fig. 3g). Thus, we concluded that LPA protects stem cells through an LPAR_1_-Gi pathway, while S1P acts mainly through an S1PR_1/3_-Gi pathway (Fig. 3h). All of the above findings could be repeated in the ethanol-induced stem cell damage model (Additional file 1: Figure S4).

### LPA and S1P prevents stem cell damage through the RAS/ERK, PI3K/Akt, and NF-κB/IL-10 pathways

**Fig. 4:**
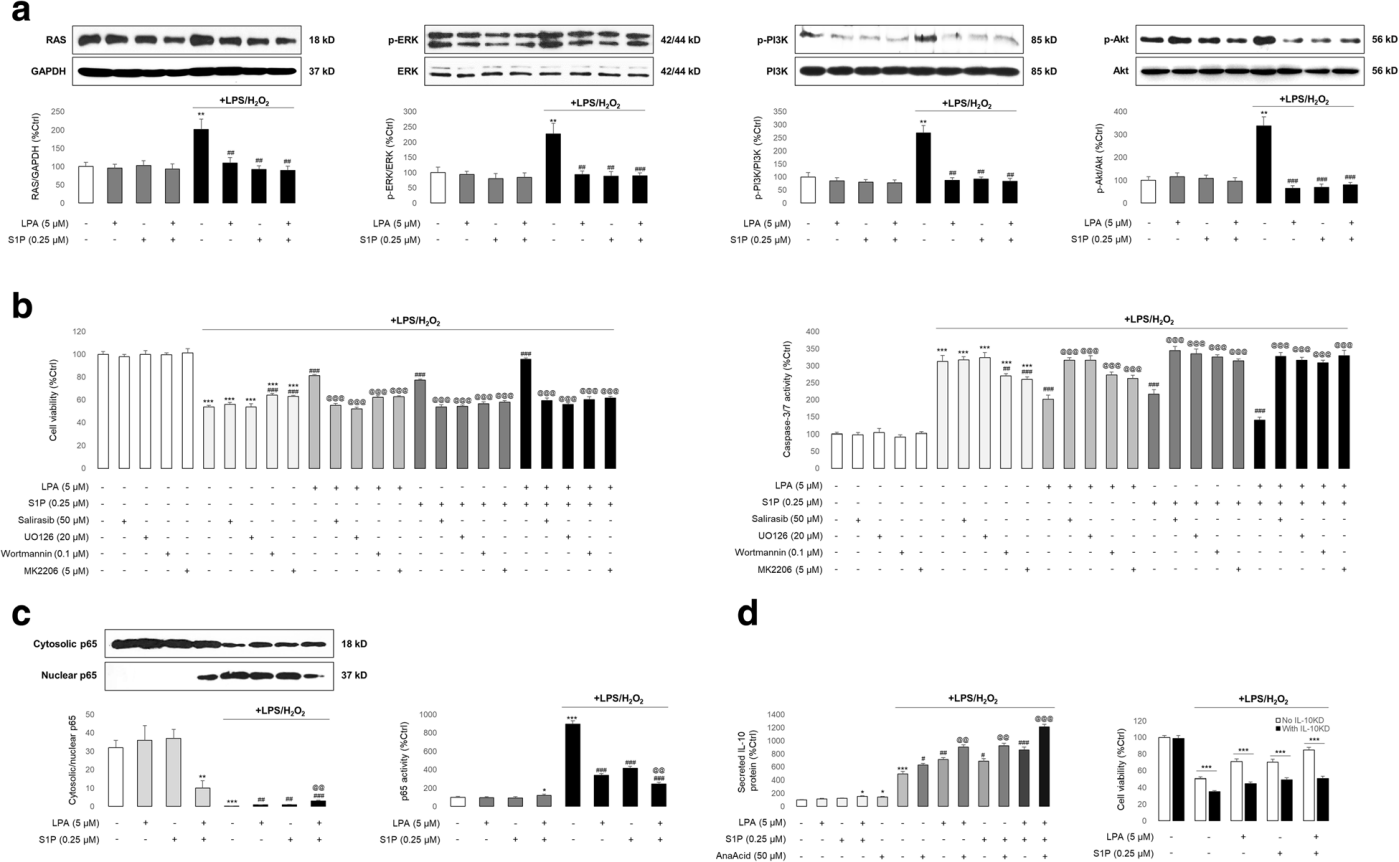
The RAS/ERK, PI3K/Akt, and NF-κB/IL-10 pathways are the downstream targets of LPAR_1_/S1PR_1/3_-mediated stem cell protection. **a** Representative images of Western blot results for RAS, phosphorylated ERK (p-ERK), total ERK, phosphorylated PI3K (p-PI3K), total PI3K, phosphorylated Akt (p-Akt), total Akt, and their quantitative data after LPS/H_2_O_2_ intoxification, in the presence or absence of LPA/S1P co-treatments (*n* = 3). **b** Changes in cell viability and caspase-3/7 activity of hADMSCs after LPS/H_2_O_2_ and LPA/S1P treatments, with or without the co-administration of salirasib (RAS inhibitor), UO126 (ERK inhibitor), wortmannin (PI3K inhibitor), or MK2206 (Akt inhibitor) (*n* = 4). **c** Changes in nuclear translocation and activation of NF-κB p65 subunit after LPS/H_2_O_2_ and LPA/S1P treatments (*n* = 3). **d** (left) Changes in IL-10 secretion of hADMSC LPS/H_2_O_2_ and LPA/S1P treatments, with or without the co-administration of NF-κB p65 inhibitor anacardic acid (AnaAcid); (right) changes in cell viability after LPS/H_2_O_2_/LPA/S1P treatments, with or without IL-10 shRNA co-transfection (*n* = 4). Data from each group are expressed as means ± SEM. Statistical comparison between groups was performed with the Kruskal–Wallis test followed by Dunn’s post-hoc test to detect differences in all groups. **p < 0.01 versus control group; ***p < 0.001 versus control group; ^##^*p* < 0.01 versus LPS/H_2_O_2_ group; ^###^*p* < 0.001 versus LPS/H_2_O_2_ group; ^@@@^*p* < 0.001 versus LPS/H_2_O_2_ + LPA or S1P group. Ctrl, control; IL, interleukin; LPA, lysophosphatidic acid; LPS, lipopolysaccharides; S1P, sphingosine-1-phosphate

Since the RAS/ERK and PI3K/Akt pathways are reported to be important in stem cell survival in harsh environments [16, 30, 31], we investigated whether those pathways were involved in LPA- and S1P-mediated cell protection. Western blot results showed that levels of RAS, phosphorylated ERK (p-ERK), p-PI3K, and p-Akt were significantly increased after treatment with LPS/H_2_O_2_, and were attenuated to control-comparable levels with LPA and/or S1P co-treatments. Total protein levels of ERK, PI3K, and Akt were not altered by any treatments performed (Fig. 4a). In addition, when salirasib (an RAS inhibitor), UO126 (an ERK inhibitor), wortmannin (a PI3K inhibitor), or MK2206 (an Akt inhibitor) were added to the cell culture medium prior to LPS/H_2_O_2_, cell viability recovery and caspase-3/7 activity inhibition by LPA and/or S1P treatments were significantly impaired, suggesting the indispensable roles of the RAS/ERK and PI3K/Akt pathways in LPA/S1P-mediated cell protection (Fig. 4b). In addition, it was demonstrated that, compared with healthy cells, LPS/H_2_O_2_ incubation significantly promoted the nuclear translocation and activation of NF-κB p65, which was inhibited by co-treatment with LPA/S1P (Fig. 4c). Since IL-10 is an important anti-inflammatory cytokine regulated by the NF-κB p65 subunit and secreted from the stem cell [32, 33], we hypothesized that the NF-κB-enhanced production of IL-10 from hADMSCs by LPA/S1P treatments was the main mechanism for the cell damage amelioration. Indeed, LPS/H_2_O_2_ incubation significantly stimulated the secretion of IL-10 from hADMSCs, while LPA and/or S1P co-treatment further enhanced its production. The application of an inhibitor of p65 (AnaAcid) exhibited an IL-10 promoting effect on the basis of LPA and/or S1P co-treatment. Importantly, when cell endogenous IL-10 was knocked down by shRNA, the cell viability recovering effects of LPA/S1P against LPS/H_2_O_2_ incubation were significantly impaired, suggesting an essential role of secreted IL-10 in autocrine/paracrine style cell protection (Fig. 4d). All results from the LPS/H_2_O_2_ model were confirmed in the ethanol-induced stem cell injury model (Additional file 1: Figure S5).

### Priming with LPA/S1P improves the therapeutic efficacy of hADMSCs in vivo

**Fig. 5:**
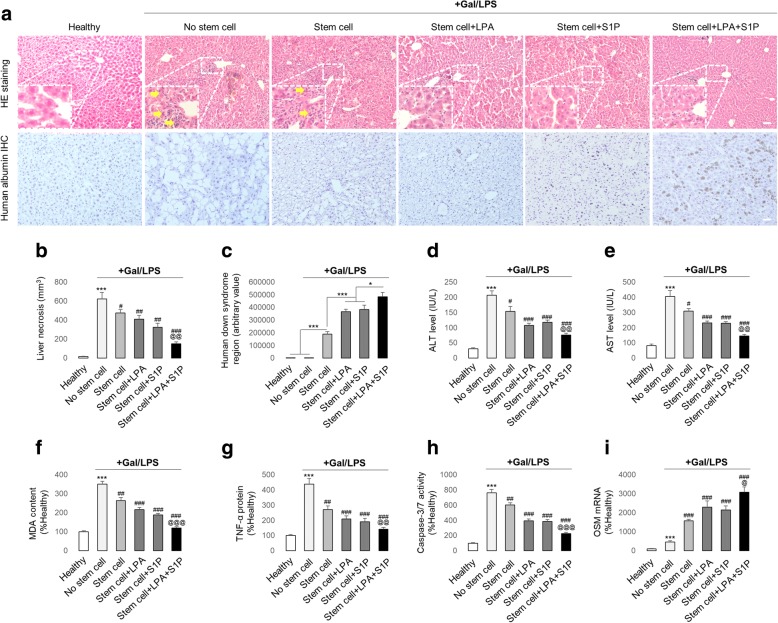
Pre-incubation with LPA and/or S1P significantly enhances the therapeutic efficacy of transplanted hADMSCs in an acute DILI model. **a** Representative images and corresponding necrotic area quantification of murine liver histology stained by H&E and IHC by human albumin antibody. Scale bar = 20 μm (*n* = 4). After Gal/LPS co-injection with or without hADMSC administration (naive or LPA/S1P treated), changes in **c** hepatic human down syndrome region, **d** serum ALT level, **e** serum AST level, **f** hepatic MDA content, **g** hepatic TNF-α protein level, **h** hepatic caspase-3/7 activity, and **i** hepatic OSM mRNA level were measured (*n* = 4). Data from each group are expressed as means ± SEM. Statistical comparison between groups was performed with the Kruskal–Wallis test followed by Dunn’s post-hoc test to detect differences in all groups. ***p < 0.001 versus healthy group; ^#^*p* < 0.05 versus Gal/LPS group; ^##^*p* < 0.01 versus Gal/LPS group; ^###^*p* < 0.001 versus Gal/LPS group; ^@^*p* < 0.05 versus Gal/LPS + LPA or S1P pre-treated stem cell transplantation group; ^@@^*p* < 0.01 versus Gal/LPS + LPA or S1P pre-treated stem cell transplantation group; ^@@@^*p* < 0.001 versus Gal/LPS + LPA or S1P pre-treated stem cell transplantation group. ALT, alanine transaminase; AST, aspartate aminotransferase; Gal, d-galactosamine; LPA, lysophosphatidic acid; LPS, lipopolysaccharides; MDA, malondialdehyde; OSM, oncostatin M; S1P, sphingosine-1-phosphate; TNF, tumour necrosis factor

To assess the possible influence of LPA/S1P treatment on stem cell transplantation efficacy, we first used an acute DILI (ALF) mouse model [7]. Three days after Gal/LPS injection, NOD/SCID mice exhibited severe hepatic injury, including hepatocyte death and inflammatory cell infiltration (Fig. 5a, b). These injuries were successfully ameliorated by hADMSC transplantation. Importantly, pre-conditioning with LPA or S1P significantly enhanced the therapeutic effects of stem cells, while combined LPA and S1P treatment showed the highest enhancing effects (Fig. 5a, b). The quantification of homed human stem cells in the murine livers by Down syndrome sequence real-time PCR, as well as the human albumin IHC test, confirmed that more stem cells survived in the injured murine liver after LPA/S1P pre-treatment (Fig. 5a, c). Moreover, changes in the murine serum ALT, AST, hepatic MDA content, TNF-α protein, and caspase-3/7 activity also reflected the effectiveness of hADMSC therapy on the DILI. Pre-treatment with LPA/S1P potently enhanced the therapeutic efficacy of stem cells on those parameters (Fig. 5d–h). Since OSM has been reported to promote liver regeneration when injury occurs, we also observed that its hepatic mRNA expression was increased after Gal/LPS challenge as an internal compensation mechanism. Stem cell transplantation enhanced the mRNA expression of OSM to accelerate the liver regeneration rate, and this process was further strengthened by pre-treatment with LPA/S1P (Fig. 5i). Collectively, our data demonstrated that transplantation with hADMSCs was an effective strategy to ameliorate Gal/LPS-induced murine liver failure, and its efficacy could be further enhanced by LPA/S1P pre-treatment.

**Fig. 6:**
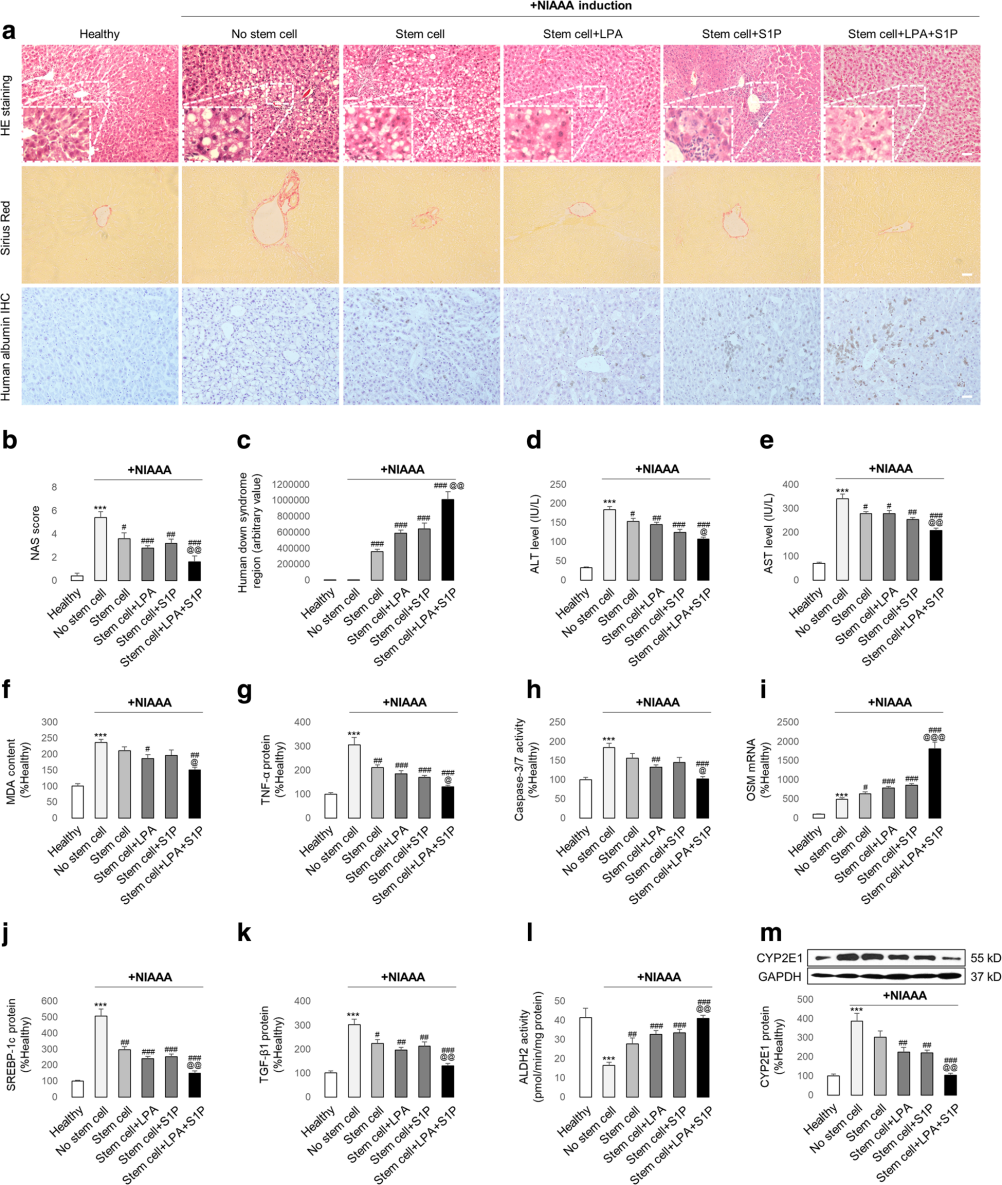
Pre-incubation with LPA and/or S1P significantly enhances the therapeutic efficacy of transplanted hADMSCs in a murine National Institute on Alcohol Abuse and Alcoholism (NIAAA) model. **a** Representative images and corresponding NAS quantification of murine liver histology stained by H&E, Sirius Red, or human albumin antibody. Scale bar = 20 μm (*n* = 4). After the establishment of a NIAAA model with or without hADMSC administration (naive or LPA/S1P treated), changes in **c** hepatic human Down syndrome region; **d** serum ALT level, **e** serum AST level; **f** hepatic MDA content; **g** hepatic TNF-α protein level, **h** hepatic caspase-3/7 activity, **i** hepatic OSM mRNA level, **j** hepatic SREBP-1c protein level, **k** hepatic TGF-β protein level, **l** hepatic ALDH2 activity (*n* = 4), and **m** hepatic CYP2E1 protein expression were measured (*n* = 3). Data from each group are expressed as means ± SEM. Statistical comparison between groups was performed with the Kruskal–Wallis test followed by Dunn’s post-hoc test to detect differences in all groups. ***p < 0.001 versus healthy group; ^#^*p* < 0.05 versus Gal/LPS group; ^##^*p* < 0.01 versus NIAAA group; ^###^*p* < 0.001 versus NIAAA group; ^@^*p* < 0.05 versus NIAAA + LPA or S1P pre-treated stem cell transplantation group; ^@@^*p* < 0.01 versus NIAAA + LPA or S1P pre-treated stem cell transplantation group; ^@@@^*p* < 0.001 versus NIAAA + LPA or S1P pre-treated stem cell transplantation group. ALDH2, aldehyde dehydrogenase 2; ALT, alanine transaminase; AST, aspartate aminotransferase; CYP2E1, cytochrome P450 2E1; Gal, d-galactosamine; LPA, lysophosphatidic acid; LPS, lipopolysaccharides; MDA, malondialdehyde; OSM, oncostatin M; S1P, sphingosine-1-phosphate; SREBP, sterol regulatory element-binding protein; TGF, transforming growth factor; TNF, tumour necrosis factor

To verify the therapeutic efficacy of hADMSCs on ALD and the influence of the LPA/S1P pre-treatment, we induced a chronic/binge ALD model using NIAAA methods. Mice with ethanol consumption exhibited typical ALD phenotypes, including fat droplet accumulation and mild inflammation and fibrosis, which could be quantified using an NAS scoring system (Fig. 6a, b). As with the results from the ALF model, transplantation with hADMSCs effectively attenuated those hepatic injuries and promoted liver regeneration (Fig. 6c–i). In addition, stem cell therapy also re-balanced hepatic lipid metabolism (SREBP-1c expression), reduced fibrosis (TGF-β expression), accelerated alcohol metabolism (ALDH2 activity), and ameliorated enzyme-mediated hepatic injury (CYP2E1 expression) (Fig. 6j–m). Pre-treatment with LPA or S1P, and particularly their combined treatment, drastically improved the therapeutic efficacy of the stem cells (Fig. 6).

### LPA/S1P pre-treated MSC therapy is safe for long-term transplantations

**Fig. 7:**
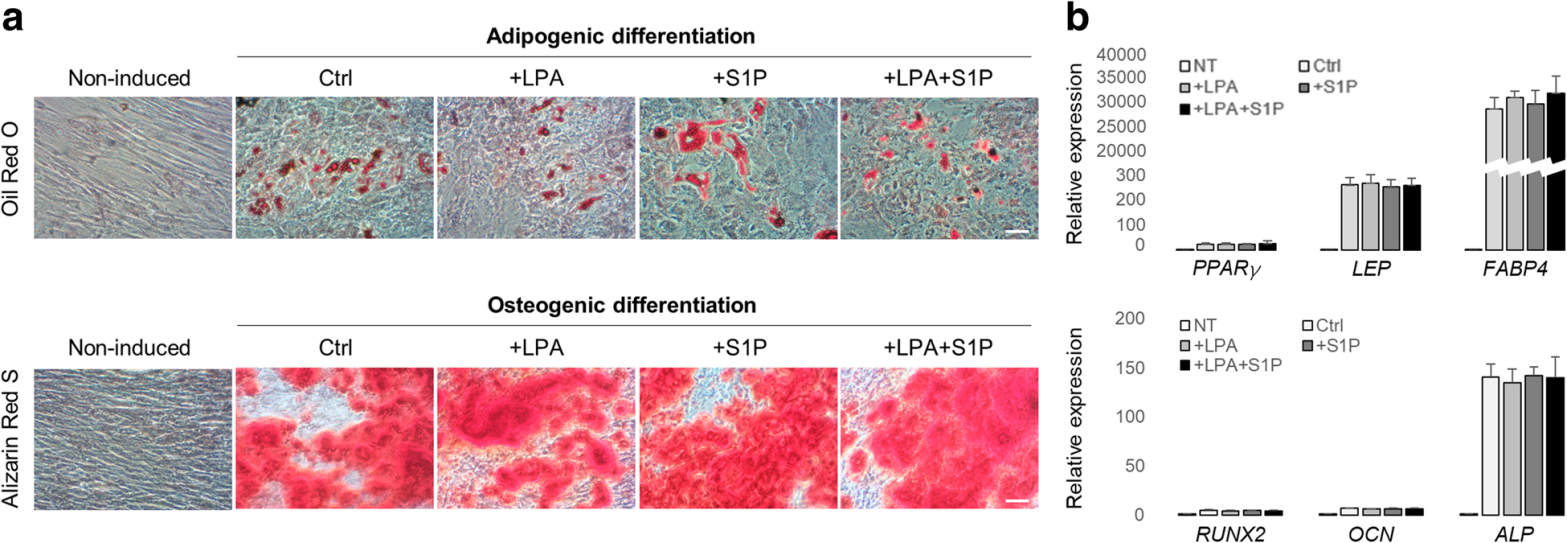
Administration with LPA and/or S1P has little effect on hADMSC adipogenic and osteogenic differentiation potential. **a** Representative images of adipogenic and osteogenic differentiation hADMSCs, after LPS/H_2_O_2_ intoxification, in the presence or absence of LPA/S1P co-treatments, characterized by Oil Red O and Alizarin Red S staining, respectively. Scale bar = 50 μm. **b** Quantitative PCR measurement for the changes in key markers of adipogenic and osteogenic differentiation, after LPS/H_2_O_2_ intoxification, in the presence or absence of LPA/S1P co-treatments (*n* = 4). Data from each group are expressed as means ± SEM. Statistical comparison between groups was performed with the Kruskal–Wallis test followed by Dunn’s post-hoc test to detect differences in all groups. ALP, alkaline phosphatase; FABP4, fatty acid binding protein 4; LEP, leptin; LPA, lysophosphatidic acid; LPS, lipopolysaccharides; NT, non-induced; OCN, osteocalcin; PPAR, peroxisome proliferator-activated receptor; RUNX2, runt-related transcription factor 2; S1P, sphingosine-1-phosphate

Safety is critically important for stem cell therapies. In addition, a pre-treatment method should not alter the multipotency of MSCs since it is critical for the tissue repair after transplantation. Thus, we tested the trans-differential potential changes in vitro and long-term transplantation tumourigenicity in vivo of LPA and/or S1P pre-conditioned hADMSCs. We found that supplementation with LPA and/or S1P did not affect the in vitro stem cell differentiation abilities into the adipogenic and osteogenic lineages, which were the most common multilineage differentiation capacity tests of MSCs, as verified by Oil Red O/Alizarin Red S staining and real-time PCR quantification of key adipogenic/osteogenic marker expressional changes (Fig. 7). Moreover, during the long-term transplantation observation period (24 weeks) in both murine hepatic injury models (ALF and ALD), no mice in any of the groups, except for the positive groups, developed tumours (i.e., tumour incidence rate was 0%). All animals in the positive groups of healthy, ALF, and ALD models showed severe symptoms of dyspnoea and minimal activity from 5 to 6 weeks after ES-3D cell injection. Gross anatomy analysis determined that 100% of those mice developed lung tumours (Table 1).

**Table 1:** Tumour incidence rate after human mesenchymal stem cell (MSCs) transplantation in healthy, acute liver failure (ALF), and alcoholic liver disease (ALD) non-obese diabetic severe combined immune-deficient (NOD/SCID) mice

Model	Test item	Injected cell number	No. of tumour-bearing mice^a^	Tumour incidence rate (%)
Healthy mice	MRC-5^b^	1 × 10^7^	0/12	0
	hADMSCs	1 × 10^7^	0/12	0
	hADMSCs with LPA/S1P^c^	1 × 10^7^	0/12	0
	ES-D3^d^	1 × 10^7^	10/10	100
ALF model	MRC-5	1 × 10^7^	0/9	0
	hADMSCs	1 × 10^7^	0/12	0
	hADMSCs with LPA/S1P	1 × 10^7^	0/15	0
	ES-D3	1 × 10^7^	9/9	100
ALD model	MRC-5	1 × 10^7^	0/12	0
	hADMSCs	1 × 10^7^	0/12	0
	hADMSCs with LPA/S1P	1 × 10^7^	0/12	0
	ES-D3	1 × 10^7^	11/11	100

hADMSC, human adipose-derived mesenchymal stem cell; LPA, lysophosphatidic acid; S1P, sphingosine-1-phosphate

^a^Dead mice during experiments were not calculated

^b^Negative control group

^c^Pre-treated with 5 μM LPA and 0.25 μM S1P for 2 h before stem cell transplantation

^d^Positive control group

## Discussion

Mesenchymal stem cell therapy has been described by a number of groups in DILI [34–36]. Instead of direct homing, transplanted stem cells primarily repair the damaged liver through paracrine mechanisms (e.g., through the secretion of anti-inflammatory and pro-regenerative factors). A recent study even demonstrated the efficacy and safety of bone marrow-derived stromal cell therapy in hepatitis B virus-related acute-on-chronic liver failure patients (cumulative survival rate: MSC 73.2% versus control 55.6%, *p* = 0.026) [37]. However, unlike DILI, basic and clinical reports for stem cell-based ALD therapy are scarce. A phase 2 trial using autologous bone marrow-derived mesenchymal stem cells to treat alcoholic cirrhosis found that stem cell transplantation safely and effectively improved histologic fibrosis and liver functions (Child–Pugh score evaluation) [38]. Since excessive cell death, oxidative stress, inflammation, and insufficient hepatic detoxification were the main characters of both DILI and ALD, we selected these models to test the transplantation efficacy and mechanism of human MSCs [39].

In addition to possible tumourigenesis, another major problem for stem cell therapy is poor therapeutic efficacy. For example, it was reported that more than 99% of the transplanted stem cells died within the first 24 h post-injection [40]. The mechanisms responsible for this phenomenon were not fully understood. Several studies have shown that severe oxidative stress, inflammation, and necrotic signals dramatically promote stem cell death. Thus, the restoration of endogenous antioxidant ability seems to be a feasible strategy to enhance the transplantation efficacy. In addition to antioxidant drug incubation, the overexpression of key antioxidant genes (e.g., Nrf2 and stromal-cell derived factor 1α) of stem cells before transplantation was shown to be another possible way to enhance efficacy [40, 41]. However, due to its drawbacks, such as viral infection-induced carcinogenesis and plasmid transfection-induced unstable overexpression, this method still needs substantial optimization before large-scale clinical applications can be considered.

The roles of LPARs and S1PRs in stem cell biology have been characterized in several reports. For example, during the progression of multiple myeloma, LPAR_1_ and LPAR_3_ transduce opposite signals to determine MSC fates as either myeloma supportive or suppressive stroma [42]. The manipulation of LPAR_3_ was found to be a novel strategy for augmenting or inhibiting the erythropoiesis of human haematopoietic stem cells [43]. The supplementation of LPA in the culture medium prevents hypoxia-induced apoptosis of bone marrow MSCs through LPAR_1_. For S1PRs, their inhibition of haematopoietic stem cell egress from the liver was found to be critical for the control of fibrosis [44]. In addition, agonism of S1PR_3_ can support haematopoietic stem cell residence and homeostasis within the bone marrow niche [45]. In a rat pulmonary artery hypertension model, S1P pre-treated hADMSCs exhibited better ameliorative properties than naive stem cells in reducing the right ventricular weight ratio and pulmonary vascular wall thickness after transplantation [46]. In the current study, we demonstrated that stimulation of LPAR_1_ or S1PR_1/3_ significantly improved the anti-stress ability and transplantation efficacy of hADMSCs in both the in vitro and in vivo DILI and ALD models. Most importantly, co-stimulation of those receptors showed the best improvement effect. We also observed that supplementation with LPA/S1P did not induce observable adverse effects, including increased cell death, change of multilineage differentiation potentials, and carcinogenesis after in vivo transplantation. Indeed, the current study has several limitations. First, additional clinical safety data, such as long-term carcinogenesis and immune-rejection, should be collected before the application of this method in patients. Second, since NOD/SCID mice have impaired T and B cell lymphocyte development and deficient natural killer (NK) cell function, the pathogenesis of ALF and ALD in these mice was different from wild-type mice because of the key roles of immune cells in those diseases.

## Conclusions

Our study demonstrates evidence that the co-stimulation of LPAR_1_ and S1PR_1/3_ increases hADMSC in-vitro anti-stress ability and in-vivo transplantation efficacy in murine DILI and ALD models. This novel strategy holds the potential to enhance the therapeutic efficacy of adult MSC-based treatment for common metabolic liver diseases.

## Additional file

Additional file 1: **Figure S1.** Optimal concurrent dose selection of LPA and S1P on LPS/H_2_O_2_- (a) or ethanol-induced (b) stem cell injury. **Figure S2.** LPA and/or S1P attenuates ethanol-induced stem cell death. (a) Changes in hADMSC viability. (b) Distribution of hADMSC apoptotic fractions. (c) Changes in hADMSC caspase-3/7 activity. **Figure S3.** LPA and/or S1P attenuates ethanol-induced stem cell oxidative stress and inflammation. (a) Quantified data of DMPO-stained signals, (b) cellular GSH/GSSG ratio, (c) cell-secreted TNF-α protein level, and (d) cell-secreted IL-6 protein level changes in hADMSC. **Figure S4.** Stimulation of LPAR_1_/Gi by LPA or S1PR_1/3_ by S1P is critical for stem cell protection. (a) Changes in cell viability and caspase-3/7 activity of hADMSCs after ethanol/LPA treatments, with or without AM966 (LPAR_1_ inhibitor), or PTX (Gi inhibitor) co-treatment. (b) with or without the W146 (S1PR_1_ inhibitor), JTE013 (S1PR_2_ inhibitor), or CAY10444 (S1PR_3_ inhibitor) co-treatment. (c) with or without PTX (Gi inhibitor) co-treatment. (d) with or without G_12/13_ shRNA co-transfection. **Figure S5.** The RAS/ERK, PI3K/Akt, and NF-κB/IL-10 pathways are the downstream targets of LPAR_1_/S1PR_1/3_-mediated stem cell protection from ethanol-induced damage. (a) Representative images of Western blot results and quantitative data. (b) Changes in cell viability and caspase-3/7 activity of hADMSCs after ethanol and LPA/S1P treatments, with or without the co-administration of salirasib (RAS inhibitor), UO126 (ERK inhibitor), wortmannin (PI3K inhibitor), or MK2206 (Akt inhibitor). (c) Changes in nuclear translocation and activation of NF-κB p65 subunit. (d) (left) Changes in IL-10 secretion ; and (right) cell viability.

## Abbreviations

Akt: Protein kinase B
ALD: Alcoholic liver disease
ALDH: Aldehyde dehydrogenase
ALF: Acute liver failure
ALT: Alanine aminotransferase
AST: Aspartate aminotransferase
CAT: Catalase
CYP2E1: Cytochrome P450 2E1
DILI: Drug-induced liver injury
DMPO: 5,5-Dimethyl-1-pyrroline-N-oxide
DMSO: Dimethyl sulphoxide
ERK: Extracellular signal regulated kinase
Gal: d-galactosamine
GAPDH: Glyceraldehyde-3-phosphate dehydrogenase
GSH: Reduced glutathione
GSSG: Oxidized glutathione
H_2_O_2_: Hydrogen peroxide
hADMSC: Human adipose-derived mesenchymal stem cell
IHC: Immunohistochemistry
IL: Interleukin
LPA: Lysophosphatidic acid
LPAR: Lysophosphatidic acid receptor
LPS: Lipopolysaccharide
MDA: Malondialdehyde
MTT: Methylthiazolyldiphenyl tetrazolium bromide
NAS: NAFLD activity score
NF-κB: Nuclear factor-kappa B
NIAAA: National Institute on Alcohol Abuse and Alcoholism
NOD/SCID: Non-obese diabetic severe combined immune-deficient
OSM: Oncostatin M
PBS: Phosphate-buffered saline
PCR: Polymerase chain reaction
PI3K: Phosphoinositide 3-kinase
PTX: Pertussis toxin
S1P: Sphingosine-1-phosphate
S1PR: Sphingosine-1-phosphate receptor
SOD: Superoxide dismutase
SREBP: Sterol regulatory element-binding protein
TGF: Transforming growth factor
TNF: Tumour necrosis factor
