# Supplementary material for: Co-stimulation of LPAR1 and S1PR1/3 increases the transplantation efficacy of human mesenchymal stem cells in drug-induced and alcoholic liver diseases
Source: Stem Cell Res Ther. 2018 Jun 14;9:161. doi: 10.1186/s13287-018-0860-y (PMC6000942; doi:10.1186/s13287-018-0860-y)
Supplement: Supplementary file 1 — Figure S1. Optimal concurrent dose selection of LPA and S1P on LPS/H2O2- (a) or ethanol-induced (b) stem cell injury. Figure S2. LPA and/or S1P attenuates ethanol-induced stem cell death. (a) Changes in hADMSC viability. (b) Distribution of hADMSC apoptotic fractions. (c) Changes in hADMSC caspase-3/7 activity. Figure S3. LPA and/or S1P attenuates ethanol-induced stem cell oxidative stress and inflammation. (a) Quantified data of DMPO-stained signals, (b) cellular GSH/GSSG ratio, (c) cell-secreted TNF-α protein level, and (d) cell-secreted IL-6 protein level changes in hADMSC. Figure S4. Stimulation of LPAR1/Gi by LPA or S1PR1/3 by S1P is critical for stem cell protection. (a) Changes in cell viability and caspase-3/7 activity of hADMSCs after ethanol/LPA treatments, with or without AM966 (LPAR1 inhibitor), or PTX (Gi inhibitor) co-treatment. (b) with or without the W146 (S1PR1 inhibitor), JTE013 (S1PR2 inhibitor), or CAY10444 (S1PR3 inhibitor) co-treatment. (c) with or without PTX (Gi inhibitor) co-treatment. (d) with or without G12/13 shRNA co-transfection. Figure S5. The RAS/ERK, PI3K/Akt, and NF-κB/IL-10 pathways are the downstream targets of LPAR1/S1PR1/3-mediated stem cell protection from ethanol-induced damage. (a) Representative images of Western blot results and quantitative data. (b) Changes in cell viability and caspase-3/7 activity of hADMSCs after ethanol and LPA/S1P treatments, with or without the co-administration of salirasib (RAS inhibitor), UO126 (ERK inhibitor), wortmannin (PI3K inhibitor), or MK2206 (Akt inhibitor). (c) Changes in nuclear translocation and activation of NF-κB p65 subunit. (d) (left) Changes in IL-10 secretion ; and (right) cell viability. [file 13287_2018_860_MOESM1_ESM.docx]

**Figure S1**


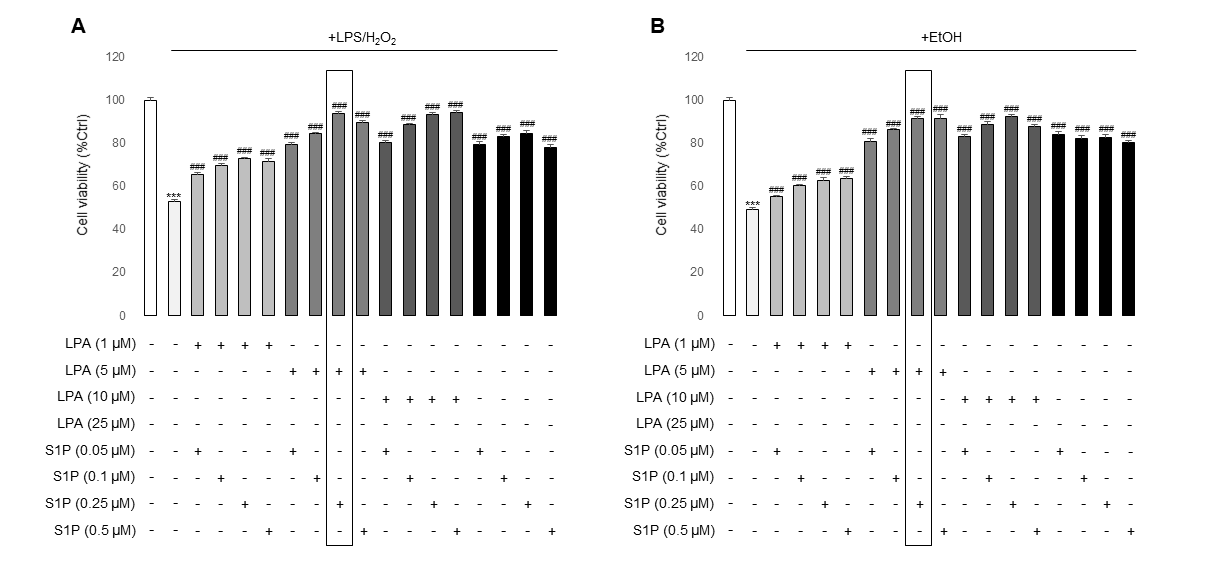


**Figure S1**. Optimal concurrent dose selection of LPA and S1P on LPS/H_2_O_2_- **a** or ethanol-induced **b** stem cell injury. Cell viability was quantified by using the MTT method. Optimal dose combinations were labeled in the figure. Data from each group were expressed as means ± SEM. Statistical comparison between groups was done using the Kruskal–Wallis test followed by Dunn’s post hoc test to detect differences in all groups. *** P < 0.001 against control group; ^###^ P < 0.001 against LPS/H_2_O_2_ or ethanol group.

**Figure S2**


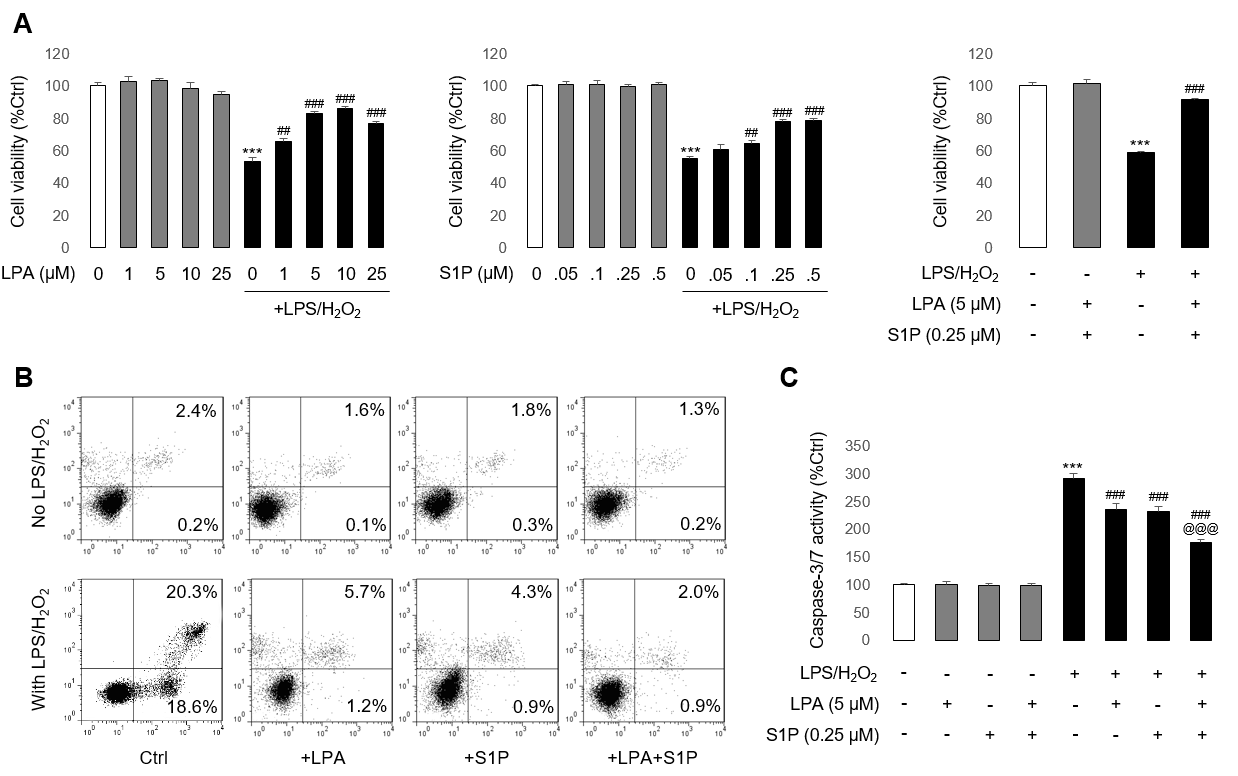


**Figure S2.** LPA and/or S1P attenuates ethanol-induced stem cell death. **a** Changes of hADMSCs viability after ethanol incubation in the presence or absence of LPA/S1P co-treatments. **b** Distribution of hADMSCs apoptotic fractions measured by flow cytometry. Upper right: late phase apoptotic ratio; Lower right: early phase apoptotic ratio. **c** Changes of hADMSCs caspase-3/7 activity after ethanol incubation in the presence or absence of LPA/S1P co-treatments. Data from each group were expressed as means ± SEM. Statistical comparison between groups was done using the Kruskal–Wallis test followed by Dunn’s post hoc test to detect differences in all groups. *** P < 0.001 against control group; ^##^ P < 0.01 against ethanol group; ^###^ P < 0.001 against ethanol group; ^@@@^ P < 0.001 against ethanol + LPA or S1P group. Abbreviations: Ctrl, control; LPA, lysophosphatidic acid; LPS, lipopolysaccharides; S1P, sphingosine-1-phosphate.

**Figure S3**


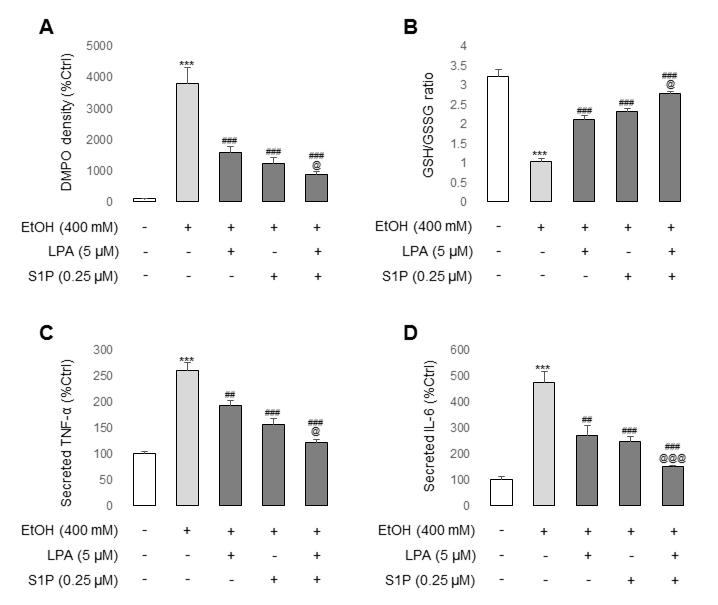


**Figure S3.** LPA and/or S1P attenuates ethanol-induced stem cell oxidative stress and inflammation. **a** Quantified data of DMPO-stained signals; **b** Cellular GSH/GSSG ratio; **c** Cell secreted TNF-α protein level; and **d** Cell secreted IL-6 protein level changes of hADMSCs after ethanol in the presence or absence of LPA/S1P co-treatments. Data from each group were expressed as means ± SEM. Statistical comparison between groups was done using the Kruskal–Wallis test followed by Dunn’s post hoc test to detect differences in all groups. *** P < 0.001 against control group; ^##^ P < 0.01 against ethanol group; ^###^ P < 0.001 against ethanol group; ^@^ P < 0.05 against ethanol + LPA or S1P group; ^@@@^ P < 0.001 against ethanol + LPA or S1P group. Abbreviations: Ctrl, control; DMPO, 5,5-dimethyl-1-pyrroline-*N*-oxide; LPA, lysophosphatidic acid; LPS, lipopolysaccharides; S1P, sphingosine-1-phosphate.

**Figure S4**


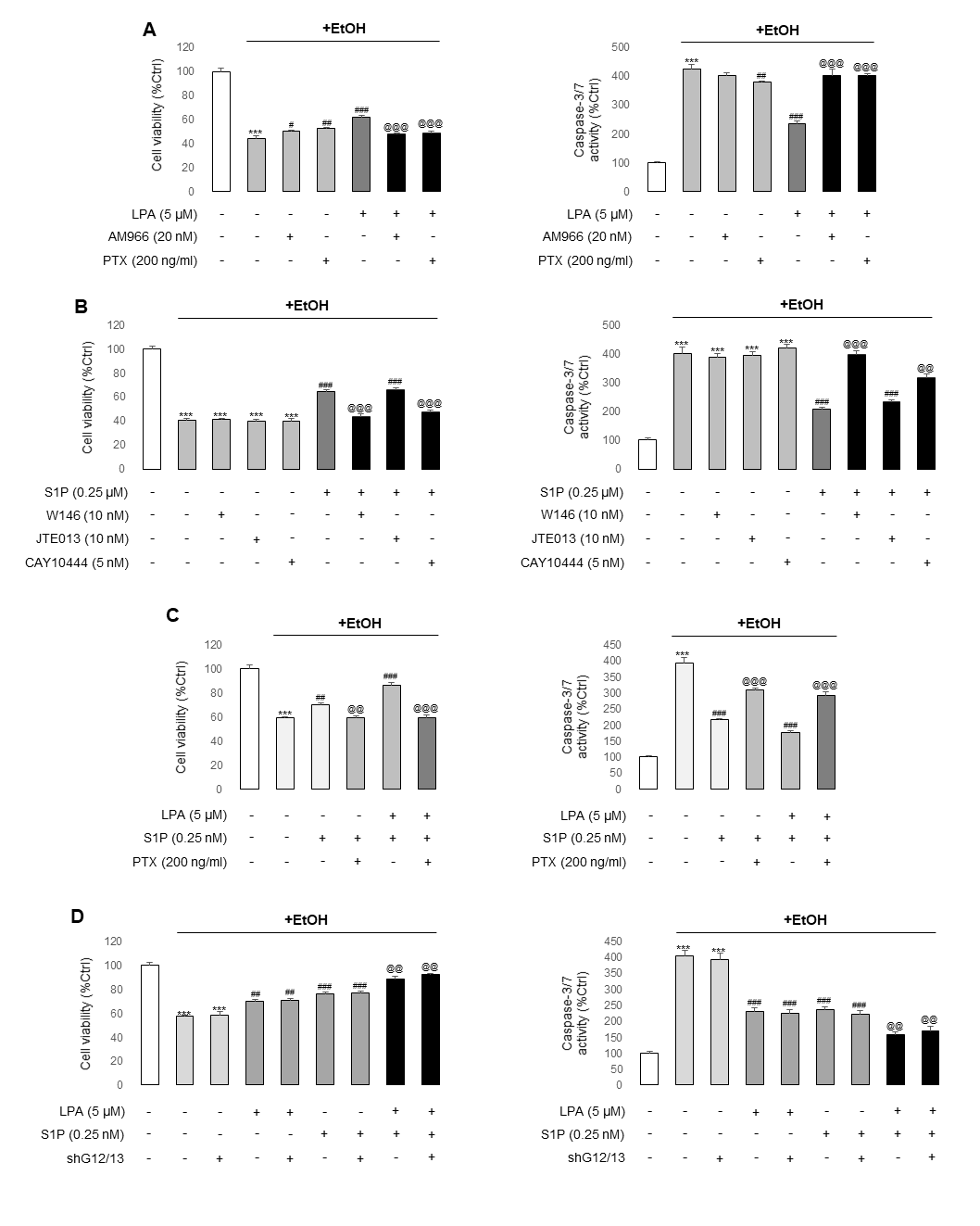


**Figure S4.** Stimulation of LPAR_1_/Gi by LPA or S1PR_1/3_ by S1P is critical for stem cell protection. **a** Changes of cell viability and caspase-3/7 activity of hADMSCs after ethanol/LPA treatments, with or without AM966 (LPAR_1_ inhibitor), or PTX (Gi inhibitor) co-treatment; **b** Changes of cell viability and caspase-3/7 activity of hADMSCs after ethanol/S1P treatments, with or without the W146 (S1PR_1_ inhibitor), JTE013 (S1PR_2_ inhibitor), or CAY10444 (S1PR_3_ inhibitor) co-treatment. **c** Changes of cell viability and caspase-3/7 activity of hADMSCs after ethanol/LPA/S1P treatments, with or without PTX (Gi inhibitor) co-treatment. **d** Changes of cell viability and caspase-3/7 activity of hADMSCs after ethanol/LPA/S1P treatments, with or without G_12/13_ shRNA co-transfection. Data from each group were expressed as means ± SEM. Statistical comparison between groups was done using the Kruskal–Wallis test followed by Dunn’s post hoc test to detect differences in all groups. *** P < 0.001 against control group; ^#^ P < 0.05 against ethanol group; ^##^ P < 0.01 against ethanol group; ^###^ P < 0.001 against ethanol group; ^@@^ P < 0.01 against ethanol + LPA or S1P group; ^@@@^ P < 0.001 against ethanol + LPA or S1P group. LPA, lysophosphatidic acid; LPS, lipopolysaccharides; S1P, sphingosine-1-phosphate.

**Figure S5**


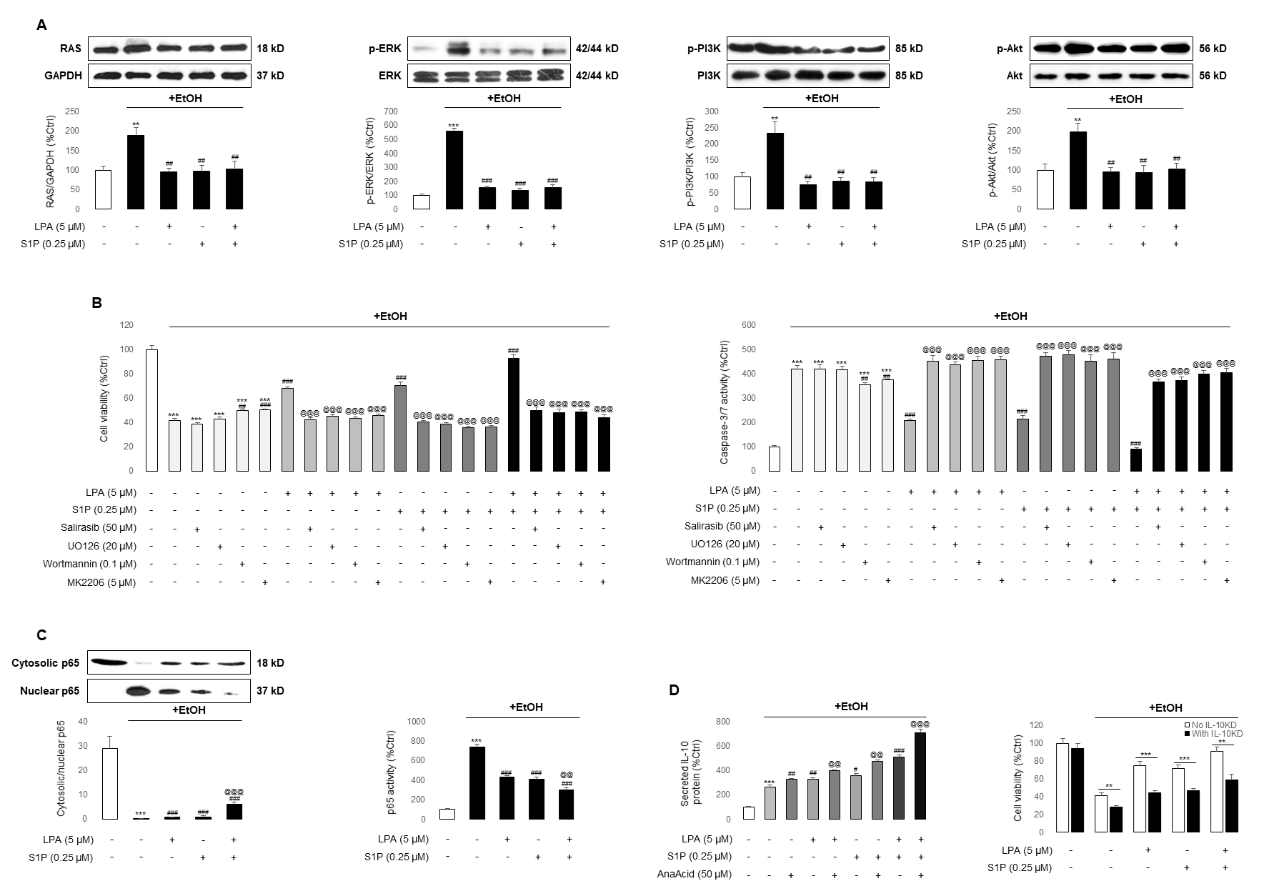


**Figure S5.** The RAS/ERK, PI3K/Akt and NF-κB/IL-10 pathways are the downstream targets of LPAR_1_/S1PR_1/3_-mediated stem cell protection from ethanol-induced damage. **a** Representative images of Western blot results for RAS, phosphorylated ERK (p-ERK), total ERK, phosphorylated PI3K (p-PI3K), total PI3K, phosphorylated Akt (p-Akt), total Akt, and their quantitative data after ethanol intoxification, in the presence or absence of LPA/S1P co-treatments (n = 3 for each group). **b** Changes of cell viability and caspase-3/7 activity of hADMSCs after ethanol and LPA/S1P treatments, with or without the co-administration of salirasib (RAS inhibitor), UO126 (ERK inhibitor), wortmannin (PI3K inhibitor), or MK2206 (Akt inhibitor). **c** Changes of nuclear translocation and activation of NF-κB p65 subunit after ethanol and LPA/S1P treatments. **d** (Left) Changes of IL-10 secretion of hADMSCs ethanol and LPA/S1P treatments, with or without the co-administration of NF-κB p65 inhibitor anacardic acid (AnaAcid); (Right) Changes of cell viability after ethanol/LPA/S1P treatments, with or without IL-10 shRNA co-transfection. Data from each group were expressed as means ± SEM. Statistical comparison between groups was done using the Kruskal–Wallis test followed by Dunn’s post hoc test to detect differences in all groups. *** P < 0.001 against control group; ^##^ P < 0.01 against ethanol group; ^###^ P < 0.001 against ethanol group; ^@@@^ P < 0.001 against ethanol + LPA or S1P group. LPA, lysophosphatidic acid; LPS, lipopolysaccharides; S1P, sphingosine-1-phosphate.
